# PRospective Observational POLIsh Study on post-stroke delirium (PROPOLIS): methodology of hospital-based cohort study on delirium prevalence, predictors and diagnostic tools

**DOI:** 10.1186/s12883-015-0351-z

**Published:** 2015-06-19

**Authors:** Elzbieta Klimiec, Tomasz Dziedzic, Katarzyna Kowalska, Aleksandra Szyper, Joanna Pera, Paulina Potoczek, Agnieszka Slowik, Aleksandra Klimkowicz-Mrowiec

**Affiliations:** Department of Neurology, Jagiellonian University Medical College, Botaniczna 3, 31-503 Krakow, Poland; Department of Neurology, University Hospital, Botaniczna 3, 31-503 Krakow, Poland

**Keywords:** Delirium, Stroke, Risk factors, Screening tools, DSM-V

## Abstract

**Background:**

Between 10 % to 48 % of patients develop delirium in acute phase of stroke. Delirium determinants and its association with other neuropsychiatric disturbances in stroke are poorly understood. The wildly accepted predictive model of post-stroke delirium is still lacking.

**Methods/design:**

This is a prospective, observational, single-center study in patients with acute phase of stroke. We aim to include 750 patients ≥18 years with acute stroke or transient ischemic attack admitted to the stroke unit within 48 hours after stroke onset. The goals of the study are: 1) to determine frequency of delirium and subsyndromal delirium in Polish stroke patients within 7 days after admission to the hospital; 2) to determine factors associated with incidence, severity and duration of delirium and subsyndromal delirium and to create a predictive model for post-stroke delirium; 3) to determine the association between delirium and its cognitive, psychiatric, behavioral and functional short and long-term consequences; 4) to validate scales used for delirium diagnosis in stroke population.

Patients will be screened for delirium on daily basis. The diagnosis of delirium will be based on DSM-V criteria. Abbreviated version of Confusion Assessment Method and Confusion Assessment Method for the Intensive Care Unit will be used for delirium and sub-delirium screening. Severity of delirium symptoms will be assessed by Delirium Rating Scale Revised 98 and Cognitive Test for Delirium. Patients who survive will undergo extensive neuropsychological, neuropsychiatric and functional assessment 3 and 12 months after the stroke.

**Discussion:**

This study is designed to provide information on clinical manifestation, diagnostic methods and determinants of delirium spectrum disorders in acute stroke phase and their short and long-term consequences. Collected information allow us to create a predictive model for post-stroke delirium.

## Background

Delirium occurs in 10 to 48 % of patients in the acute phase of stroke [1]. Delirious patients have almost 5-times higher risk of both in hospital and twelve-month mortality than non-delirious ones [2]. Patients with post-stroke delirium (PSD) are also over three times more likely to be discharged to long-term care institutions or nursing home and their hospital stay is on average 9 days longer [2]. Compared to non-delirious stroke patients, delirious patients have worse functional outcome after the stroke [3, 4] and lower quality of life one-month after hemorrhagic stroke [5]. Patients who are delirious in the acute phase of stroke have lower score in Mini Mental State Examination 1, 6 and 12 months after the stroke [6] and increased risk for dementia two years after the stroke [7].

A number of predisposing factors for delirium in stroke were identified. The leading factors in stroke include older age and the presence of infection [3, 8, 9]. Stroke-related risk factors include greater stroke severity [3], stroke in anterior circulation [3, 10] and specific symptoms of stroke such as aphasia, neglect or hemianopia [4, 8, 11]. The impact of lesion location and stroke type is uncertain [4, 10–12]. The predisposing role of environmental factors has not been investigated in stroke, however in non-stroke acute settings, only the use of restrains was significantly associated with higher risk of delirium [13].

Delirium as a life threating process requires quick identification of affected patients. Therefore, simple and reliable diagnostic methods are needed. New diagnostic criteria for delirium were proposed in the fifth edition of Diagnostic and Statistical Manual of Mental Disorders (DSM-V) [14, 15]. The concordance between commonly used screening tools and new criteria was not tested in stroke settings.

Recently, Oldenbeuving *et al.* conducted an analysis on 800 patients with ischemic stroke and described a tool to predict the risk for PSD; the score is based on patients age, stroke severity, stroke subtype and infection [12]. This model has a sensitivity of 76 % and specificity of 81 % for PSD diagnosis. The usefulness and generality of this model needs verification in other independent studies since it was derived and validated in two stroke units in the Netherlands.

Neuropsychiatric syndromes including depression, apathy, anxiety, emotional liability, fatigue and aggression are common after stroke. Depression occurs more frequently in patients after stroke than in general population. The proportion of people with depression ranges between 29 % [16] and 33 % [17] up to one year after stroke. Anxiety after stroke also occurs frequently: meta-analyses from 44 published studies, showed the overall estimate of anxiety disorders diagnosed by clinical interview was 18 % and was 25 % for anxiety assessed by rating scale between 1 to 6 month after stroke [18]. Fatigue 15 months after stroke was found in 57 % of patients [19]. In meta-analysis from 24 studies, apathy was detected in 34.6 % of patients 3 months after stroke [20].

The cause of these syndromes is unknown but their frequent overlap suggests common underlying mechanisms i.e. fluctuations in neurotransmitters, inflammatory cascade or disrupted functional connectivity in the limbic system [21]. The association of these neuropsychiatric syndromes with delirium were not explored.

Post stroke delirium is a multifactorious, life treating process, still poorly understood. New, prospective studies are needed to identify patients at risk of PSD for future trials of preventive strategies

## Methods/design

### Study design

PRospective Observational POLIsh Study on Post-Stroke Delirium (PROPOLIS) is observational, prospective, single center, hospital-based cohort study conducted in Department of Neurology, Jagiellonian University Medical College, Krakow, Poland. The frequency of post-stroke delirium spectrum disorders, risk factors, diagnostic tools and prognosis in consecutive 750 stroke patients admitted to the hospital within 48 hours after the stroke onset will be investigated.

- The Local Bioethics Committee approved the study (KBET/63/B/2014).
- The Leading National Research Centre funded the collection of data for the study.

### Objectives

The goals of PROPOLIS are: 1) to determine frequency of delirium and subsyndromal delirium occurring within 7 days after admission to the hospital in Polish stroke patients; 2) to determine factors associated with incidence, severity and duration of delirium and subsyndromal delirium and to create a predictive model for PSD; 3) to determine the association between delirium and its cognitive, psychiatric, behavioral and functional short and long-term consequences; 4) to validate methods used for delirium diagnosis in stroke population.

### Study population

#### Patients

All consecutive patients ≥18 years with acute stroke or transient ischemic attack (TIA) admitted to the Stroke Unit within 48 hours after stroke onset will be eligible to participate in the study.

#### In-hospital procedures

Detailed inclusion and exclusion criteria are shown in Table 1.

**Table 1:** Inclusion and exclusion criteria for the study

Inclusion criteria	Exclusion criteria
• Age ≥ 18 years	• Coma
• Acute stroke or transient ischemic attack	• Patients with brain tumor
• Admission to the hospital within 48 hours of symptoms onset	• Alcohol withdrawal delirium
• Language: Polish	• Cerebral venous thrombosis
• Written informed consent by patient or legal guardian	• Subarachnoid hemorrhage
	• Trauma
	• Diseases with life expectancy less that one year e.g. malignancy
	• Vasculitis

All eligible patients will be screened for delirium on daily basis. Baseline assessment performed during the first ten days of hospitalization consists of five elements: 1) collection of data about predisposing factors obtained from patients and their families; 2) daily basis delirium assessment during the first seven days after admission; 3) cognitive screenings performed between days 1–2 and 7–10 after admission; 4) evaluation of emotional and behavioral disturbances between 7–10 day of hospitalization.

#### Predisposing factors assessment

Data about three groups of potential predisposing factors will be collected: socio-demographic, medical and stroke-related. Table 2 shows the details.

**Table 2:** Instruments for predisposing factors assessment

Predisposing factors	Instrument
Socio-demographic	Age
	Gender
	Education
Medical condition	
Medication	Anticholinergic Risk Scale [25]
Pre-stroke functional status	Modified Ranking Scale [47], Instrumental Activities of Daily Living [48]
Health history	Cumulative Illness Rating Scale [49]
Loss of visual acuity, hearing impairment	Medical history, presence of glasses, hearing devices
Infections (pneumonia, urinary tract infection)	Diagnosis based on criteria from U.S. Centers for Disease Control and Prevention [50]
Biochemical disturbances	sodium <136 or >145 mmol/L, potassium <3.5 or >5.1 mmol/L, glucose <3.3 or >5.6 mmol/L, urea nitrogen >8,07 mmol/L, creatynine >106 mmol/L
Pre-stroke dementia	Informant Questionnaire on Cognitive Decline in the Elderly [23]
Pre-stroke behavioral and emotional disturbances	Neuropsychiatric Inventory [37]
Stroke characteristic	
Stoke severity	National Institutes of Health Stroke Scale [51]
Subtypes of ischemic stroke	The Oxford Community Stroke Project classification [52]
	Trial of Org 10172 in Acute Stroke Treatment classification [53]
Stroke symptoms (aphasia, neglect, hemianopia)	Clinical examination

The assessment of pre-existing dementia will be conducted within 48 hours of stroke onset using a Polish translation of the Informant Questionnaire on Cognitive Decline in the Elderly (IQCODE), version validated in Polish population [22]. The IQCODE is 26-item questionnaire, which asks an informant to rate degree of change over a ten-year period in various aspects of patient’s memory and other intellectual abilities. It relies on the reports of informant and obtains historical material, allowing an assessment of cognitive decline rather than current cognitive impairment. The items are rated on 5-point scale from 1 - much improved to 5 - much worse [23]. The cut- off score for presence of pre-stroke dementia will be 3.4 [24]. For anticholinergic burden assessment the scale proposed by Carnahan will be used, because positive voice shows good correlation with serum anticholinergic activity [25].

#### Delirium assessment

Abbreviated version of Confusion Assessment Method (bCAM) for verbal patients and Intensive Care Units version (CAM-ICU) will be used for non-verbal patients [26–28] for delirium screening. Severity of delirium symptoms will be assessed by Delirium Rating Scale Revised 98 (DRS-R–98) [29] and Cognitive Test for Delirium (CTD) [30]. CTD rates symptoms at the time of administration, while DRS and DRS-R–98 rates the preceding 24 hours. Delirium Motor Checklist (DMC) [31]will be used to assess number of hyper- and hypoactive symptoms. Delirious patients will be divided into hypo-, hyperactive, mixed and no subtype according to criteria from Delirium Motor Subtype Scale 4 [32]. Short, structured questionnaire regarding night and day behavior and cognitive fluctuations will be fulfilled by ward nurses. The diagnosis of delirium will be made taking into account clinical observation, information from structured nurses questionnaire and cognitive tests. We will use diagnostic criteria for delirium according to DSM-V [33]. If patients are not able to perform cognitive evaluation, the diagnosis will be based only on observation, structured nurses questionnaire and DSM-V criteria for delirium.

#### Subsyndromal delirium assessment

Subsyndromal delirium will be diagnosed when one or more new bCAM/CAM-ICU core symptoms, that did not meet CAM/CAM-ICU criteria for delirium and do not progress to delirium, will be present [34].

#### Cognitive assessment

The Montreal Cognitive Assessment (MoCA)[35], Frontal Assessment Battery (FAB) [30] and Cognitive Test for Delirium [36] will be used to assess cognitive functions.

#### Emotional and behavioral assessment

We will gather the information from the spouse/caregiver regarding pre-stroke behavioral functioning on Neuropsychiatric Inventory [37]. The presence of depressive symptoms will be assessed by Patient Health Questionnaire (PHQ–9) [38]; apathy by Apathy Evaluation Scale [39]; anxiety by State Trait Anxiety Inventory [40] and aggression by The Buss-Durke Inventory [41].

### Follow up-measures

Patients will undergo neurological, functional, cognitive, emotional and behavioral assessment 3 and 12 months after the stroke. The assessment will be done by a neurologist and psychologist who are blind to patient status at baseline.

Functional status will be assessed by Instrumental Activities of Daily Living (IADL) and modified Rankin Scale (mRS).

Neuropsychological examination will be performed by a psychologist. The global cognitive functioning will be assess by MoCA test [30]. On neuropsychological examination the following cognitive functions will be assessed: frontal executive functions (category and letter fluency, subtests from Mattis Dementia Rating Scale (MDRS) [42], Trail Making Tests part A and B [43], FAB [36]), orientation (orientation items of MoCA test), attention and concentration (digit span, subtest of MoCA, Digit and Letter Cancelation Test [44]), reasoning (calculation, arithmetic and verbal problem solving), constructional and visuospatial functions (subtests of MDRS, construction of MoCA, Rey-Osterrieth Complex Figure Test) [44], verbal memory (Luria’s Verbal Learning Test [44]), nonverbal memory (Rey-Osterrieth Complex Figure Test), language (repetition, naming), gestural praxis (subtests of MDRS, pantomiming of object use without objects), gnosis (identification of objects and naming of objects’ pictures), writing (subtests of MDRS).

Patients with at least 2 points difference between the initial and follow up examinations (with a higher score at follow up), will be diagnosed with transient cognitive impairment [45].

To track the course of emotional and behavioral disturbances we will use the same scales as for baseline assessment; PHQ–9, Apathy Evaluation Scale, State-Trait Anxiety Inventory and The Buss-Durke Inventory.

If patients are not willing or are not able to attend follow up visit they and/or their care-givers will be interviewed via telephone. Patients if eligible, will undergo the telephone version of MoCA, the cut off for cognitive decline diagnosis will be 18 points [46]. The telephone interview with a care-giver will include functional evaluation (Rankin Scale, IADL), cognitive functions evaluation (IQCODE), depression (NPI items for depression) and structured questionnaire regarding recurrent stroke, placement in nursing home and mortality. The cut off score on IQCODE for post-stroke dementia will be 3.4 [24].

**Fig. 1:**
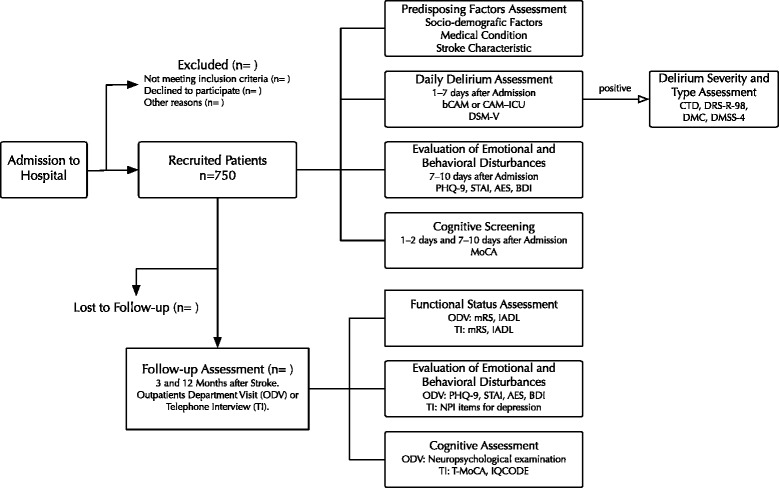
Flowchart for the study procedures. AES–Apathy Evaluation Scale, bCAM–Abbreviated version of Confusion Assessment Method, BDI–The Buss-Durke Inventory, CAM-ICU–Intensive Care Units Version of Confusion Assessment Method, CTD–Cognitive Test for Delirium, DMC–Delirium Motor Checklist, DMSS-4–Delirium Motor Subtype Scale 4, DRS-R-98–Delirium Rating Scale Revised 98, DSM-V–The Fifth Edition of Diagnostic and Statistical Manual of Mental Disorders, IADL–Instrumental Activities of Daily Living, IQCODE–Informant Questionnaire on Cognitive Decline in the Elderly, MoCA–Montreal Cognitive Assessment, mRS–modified Rankin Scale, Neuropsychological examination–described in the text, NPI–Neuropsychiatric Inventory, PHQ-9–Patient Health Questionnaire, Predisposing Factors Assessment–described in the Table 2., STAI–State Trait Anxiety Inventory, T-MoCA–telephone version of Montreal Cognitive Assessment

For those patients who are not able to undergo the telephone interview only the care-giver will be interview according to the same procedure as described above. Study plan flow chart is shown in Fig. 1.

### Data monitoring body

Collection of the data will be performed by trained raters: psychologists and neurologists. Initial diagnosis of delirium will be reassessed by experienced senior neurologists and neuropsychologist.

### Statistical analysis

To identify predisposing factors multivariable logistic regression analyses will be performed for delirium and subsyndromal delirium separately. Previously published predisposing factors will be investigated first, afterward other factors including confounders and analyzing effect-measure modifications will be investigated. Potential risk factors will be narrowed along axes using the following criteria: (1) prevalence of at least 5 % for discrete variables; (2) relative risk of 1.3 or higher for delirium at discharge in bivariable analyses (or a statistically significant parameter estimate at P = .10 for continuous variables) and (3) clinical relevance. To exclude variables of multicollinearity, pairwise correlations between the potential explanatory variables will be examined. Final independent risk factors will be defined with backward elimination procedure. To evaluate effects of the predictors on delirium severity and delirium length, a multivariate linear regression adjusted for the covariates and its interactions mentioned above model will be used. The prognostic ability of the model to predict delirium will be calculated using the area under the receiver operating characteristics curve ranging from 0.5 (no discrimination above chance) to 1.0 (perfect discrimination). To assess relationship between delirium and cognitive impairment, psychiatric and behavioral disturbances Student’s *t*-test, Mann–Whitney and chi-square tests will be calculated as appropriate. The significance level will be α =0,05 for two-sided null hypotheses.

Validity of bCAM for verbal and CAM-ICU for non-verbal patients will be assessed by calculation of sensitivity and specificity compared with the reference standard (DSM-V criteria), using standard formula with 95 % confidence intervals.

## Discussion

Delirium is a serious complication after stroke. PROPOLIS aims to investigate the frequency of delirium spectrum disorders in Polish population, their risk factors and long- and short-term prognosis. It also aims to validate delirium screening tools against DSM-V criteria.

The strong points of our study are 1) relatively large number of enrolled, unselective stroke patients including those with both ischemic and hemorrhages stroke, TIA and patients with dysphasia; 2) daily screening for delirium; 3) standardized evaluation of cognitive and behavioral correlates.

PROPOLIS will have a clearly defined, comprehensive delirium assessment based on a new diagnostic criteria DSM-V.

Neuropsychiatric aspects of behavior in patients with stroke will be studied, in their connection with delirium and consequences for short- and long-term prognosis.

Delirium is a complex process requiring input from many levels including identification of the patients at risk, control modifiable risk factors and introduce pharmacological and non-pharmacological treatment as soon as possible.

PROPOLIS is designed to prospectively investigate delirium spectrum disorders in large Polish stroke population, in the interest of the future patients. We hope, the study will allow us to build a neuro-behavioral predictive model of PSD spectrum disorders.
